# Residual Vein Thrombosis After Deep Vein Thrombosis in Patients Treated with DOACs: Incidence and Associated Factors

**DOI:** 10.3390/jcm14175991

**Published:** 2025-08-25

**Authors:** Marco Bardetta, Matteo Simoncini, Federica Valeri, Andrea Pizzuto, Cristina Dainese, Carola Sella, Annamaria Porreca, Benedetto Bruno, Alessandra Borchiellini

**Affiliations:** 1 Regional Centre for Hemorrhagic and Thrombotic Diseases, AOU Città della Salute e della Scienza, 10126 Turin, Italy; marco.bardetta@aslvco.it (M.B.);; 2 Division of Hematology, AOU Città della Salute e della Scienza, 10126 Turin, Italy; benedetto.bruno@unito.it; 3 Department of Molecular Biotechnology and Health Sciences, University of Turin, 10126 Turin, Italy; 4 Division of Internal Medicine 2, AOU Città della Salute e della Scienza, 10126 Turin, Italy; 5 Department of Human Sciences and Promotion of the Quality of Life, IRCCS San Raffaele Roma Open University, 00166 Rome, Italy; 6 Unit of Clinical and Molecular Epidemiology, IRCCS San Raffaele Roma, 00166 Rome, Italy

**Keywords:** direct oral anticoagulants, thrombosis, recanalization, residual vein obstruction, residual vein thrombosis

## Abstract

**Background/Objectives:** After an initial course of anticoagulation for deep vein thrombosis (DVT), identifying patients at higher risk of recurrence remains a clinical challenge. The role of residual vein thrombosis (RVT) in this setting is still debated, as most available evidence derives from retrospective studies or from the Warfarin era. We conducted a study to evaluate the incidence of RVT in patients treated with direct oral anticoagulants (DOACs) and to identify the clinical factors associated with its persistence. We also compared the outcomes from the two most prescribed drugs in Italy, Apixaban and Rivaroxaban. **Methods:** A total of 113 patients with newly diagnosed DVT underwent follow-up visits at 6 weeks (T1), 3 months (T2) and 6 months (T3) after diagnosis. RVT was assessed by compression ultrasonography and clinical, family and pathological history data were collected. Ninety-six patients were included in the final statistical analysis. **Results:** RVT was detected in 68.2%, 52.1% and 37.7% of patients at T1, T2 and T3, respectively. Factors significantly associated with RVT at T2 were male sex, femoral vein involvement and a family history of DVT. No significant differences were observed between Apixaban and Rivaroxaban. Prior episodes of thrombosis, smoking, diabetes and obesity were not associated with RVT at 3 months. **Conclusions:** Our findings confirm that RVT rates progressively decrease over time, as previously observed in the Coumarins era, but suggest a stronger early response to DOACs, particularly during the first three months of therapy. Moreover, DOACs appear to provide more effective protection in patients with risk factors for venous disease.

## 1. Introduction

Long-term anticoagulation refers to extending treatment beyond the standard 3-6 months of primary therapy, which is internationally accepted as the standard of care for newly diagnosed deep vein thrombosis (DVT) or pulmonary embolism (PE) [1]. The rationale of this strategy is to prevent recurrences in patients at higher risk, particularly after an unprovoked episode [2,3].

However, this approach is challenging because each patient has individual characteristics that define a unique balance between thrombotic and bleeding risk. Physicians must determine whether this balance tips toward one side or the other. Long-term anticoagulation should only be considered when thrombotic risk outweighs bleeding risk, and this assessment must be periodically reassessed over time. Conversely, when hemorrhagic risk factors are predominant (e.g., advanced age, severe renal or hepatic impairment, cancer, diabetes), therapy should be as short as possible, with 3 months generally preferred over longer durations [4].

The assessment of recurrence risk has long been debated. Although several predictive scores have been validated, their accuracy remains uncertain, particularly in the era of direct oral anticoagulants (DOACs) [5]. Multiple factors are recognized as predisposing to venous thromboembolism (VTE), such as smoking, diabetes, hypertension, immobility, autoimmune and inflammatory diseases, surgery and antiphospholipid syndrome. Other variables, including sex, body mass index, hormonal therapy, varicose veins, cancer and genetic thrombophilia, have been incorporated into recurrence prediction models [6,7]. For instance, smoking is associated with an increased relative risk of VTE, with a dose–response effect for every additional ten cigarettes smoked per day [8]. 

Beyond clinical variables, laboratory and imaging findings are also considered in risk stratification. Among these, the D-dimer assay has demonstrated acceptable prognostic value for predicting recurrence [9]. Residual vein thrombosis (RVT), also referred to as residual vein obstruction (RVO), assessed by compression ultrasonography (CUS), has gained attention as a potential predictor of recurrence when specific criteria are met, particularly within the first three months after diagnosis, when its predictive value appears strongest [10,11,12]. Nonetheless, the routine use of RVT is limited by methodological heterogeneity, a lack of standardized measurement, the variable timing of assessment and conflicting results regarding its prognostic value [13,14,15,16]. Furthermore, most prior studies included patients treated with conventional anticoagulants (i.e., Coumarins, heparins and Fondaparinux). Only a few investigations have explored the effects of DOACs on thrombus resolution and recanalization after DVT, and these were often limited by small sample sizes and short follow-up periods [17,18]. In 2017, a multicenter study by Prandoni and colleagues reported significantly lower RVT rates in patients treated with DOACs compared with those receiving vitamin K antagonists (VKAs) [19]. Overall, data on how DOACs influence recanalization rates remain limited, as do insights into factors that may predict the persistence of RVT. 

The present study, named READ (“residual vein thrombosis and DOACs”), was designed to assess and compare the efficacy of DOACs in promoting recanalization and to evaluate their impact on risk factors for RVT. Specifically, the primary endpoint was the ultrasonographic assessment of RVT at three time points: six weeks (T1), three months (T2) and six months (T3) after a new diagnosis of lower-limb DVT. Secondary endpoints included the identification of predictors associated with RVT at T2, as well as a head-to-head comparison of the two most commonly prescribed DOACs in Italy—Rivaroxaban and Apixaban—in terms of recanalization.

## 2. Materials and Methods

### 2.1. Study Design

READ is a spontaneous, prospective, observational, single-center cohort study. Patient enrolment began in November 2019 and was completed in June 2023. Inclusion criteria were as follows:

▪ Male or female aged ≥ 18 years old;
▪ A recent, radiologically confirmed, uni- or bilateral lower-limb DVT involving the common femoral vein and/or the popliteal vein (the presence of PE did not preclude enrolment);
▪ At least 2 years elapsed since the previous DVT, if the current episode was an ipsilateral recurrence;
▪ Indication for anticoagulation treatment;
▪ Eligibility for DOACs therapy, initiated within 45 days of diagnosis;
▪ Enrolment within 3 months of diagnosis.

Exclusion criteria were failure to meet one or more of the above criteria; new DVT occurring under ongoing full-dose anticoagulation; ipsilateral recurrence occurring more than 2 years after the first event in the presence of ultrasonographically documented RVT; absolute contraindications to DOAC therapy; or initiation of anticoagulation more than 15 days after diagnosis. 

Since the primary endpoint was the evaluation of RVT at 3 months, enrolment was allowed up to T2.

### 2.2. Baseline Assessment

At the first clinical evaluation, the following data were recorded: demographic characteristics and habits (e.g., sex, age, weight, smoking status), thrombosis-related features (site, extension, concomitant PE or thrombosis at other sites), personal and family risk factors for VTE, bleeding risk factors and relevant comorbidities (e.g., hypertension requiring treatment, diabetes, recent surgery, acquired or genetic thrombophilia, inflammatory diseases). Laboratory parameters were also collected, including hemoglobin, platelet count, serum creatinine and transaminase levels. DVT was classified as provoked or unprovoked according to ISTH recommendations [20].

### 2.3. Ultrasonographic Assessment

At each visit, CUS was performed using a linear probe at two sites of each affected limb:

1. The common femoral vein at the groin level, from the confluence of the deep and superficial femoral veins to the great saphenous vein;
2. The popliteal vein, from its proximal tract to the confluence of the calf veins.

The transverse venous diameter was measured and RVT was defined as an incompressibility ≥ 4 mm, according to the 2017 criteria of Prandoni et al. [21]. Each patient underwent two consecutive ultrasonographic examinations performed independently by two sonographers, who documented their findings on standardized forms.

### 2.4. Clinical Outcomes

Potential thrombotic recurrences and bleeding events were recorded. Bleeding episodes were classified as major or clinically relevant non-major bleedings (CRNMB) according to ISTH criteria [22]. As recommended by the American College of Chest Physician (ACCP) guidelines, a bleeding risk score was calculated for each patient [23].

### 2.5. Statistical Analysis

Descriptive analysis for quantitative variables was performed reporting the second quartile (median) as the measure of central tendency, and the first and third quartiles (Q1, Q3) as measures of variability. Qualitative variables were summarized as absolute frequencies and percentages. Univariate associations between categorical variables, including the evaluation of potential predictors of RVT, were assessed using Pearson’s χ^2^ test. Differences between quantitative variables across groups were assessed using the Mann–Whitney U test. A multivariable logistic regression was performed to assess the predictive power of factors that were statistically significant in the univariate analysis. Odds ratios (OR) with 95% confidence intervals (CI) and *p*-values are reported. Statistical significance was set at *p* ≤ 0.05. All analyses were performed using R statistical software (version 4.2).

## 3. Results

Among the 113 consecutive patients recruited, 17 were excluded from the statistical analysis because they were either lost to follow-up before the T2 assessment or required a switch from DOACs to heparins or VKAs due to newly arising clinical conditions. Consequently, demographic, clinical, laboratory and radiological data from 96 patients were included in the analysis. Since some patients were enrolled after six weeks from DVT diagnosis, the number of participants at T1 was lower than at T2. Conversely, due to additional losses to follow-up after T2, the number at T3 was smaller. Overall, 85 patients were assessed at T1, 96 at T2 and 77 at T3.

Of the 96 patients analyzed, 63 were men (65.6%) and the mean age was 61.9 ± 16.9 years, with more than half (57%) aged over 60 years. 

Seventeen patients (17.7%) reported a family history of thrombosis and six of them were found to carry inherited thrombophilia. Overall, 55 patients underwent thrombophilia testing, with 40% testing positive for genetic abnormalities or antiphospholipid antibodies.

With regard to personal history, 10% had a previous episode of DVT. Twenty patients (20.8%) were current smokers and 20 (20.8%) reported alcohol abuse (≥ 8 units per week). Nine patients (9.4%) had active cancer, 7 (7.3%) had diabetes, 3 (3.1%) had chronic liver disease, 17 (17.7%) had chronic renal failure and 47 (48.9%) had hypertension requiring treatment.

Concerning the site of thrombosis, 52% involved the popliteal vein alone, 8% the femoral vein alone and 39% both veins; in 7.3% of cases, thrombosis was bilateral. Concomitant PE occurred in 37.5% of patients.

More than two-thirds of patients were diagnosed within one month of symptoms onset, whereas more than 15% were diagnosed after at least four weeks. Twelve patients were asymptomatic, and their diagnosis was incidental.

Based on etiological evaluation, 44 patients (45.8%) were classified as having unprovoked DVT, 31 (32.3%) had DVT associated with weak risk factors, 12 (12.5%) with strong risk factors and 9 (9.4%) with cancer-associated thrombosis.

Treatment distribution was as follows: 50 patients (52.1%) received Apixaban, 42 (48.9%) Rivaroxaban, 3 (3.1%) Edoxaban and 1 (1.0%) Dabigatran.

Table 1 and Table 2 summarize the main characteristics of the patients included in the statistical analysis.

### 3.1. Primary Endpoint

**Figure 1:**
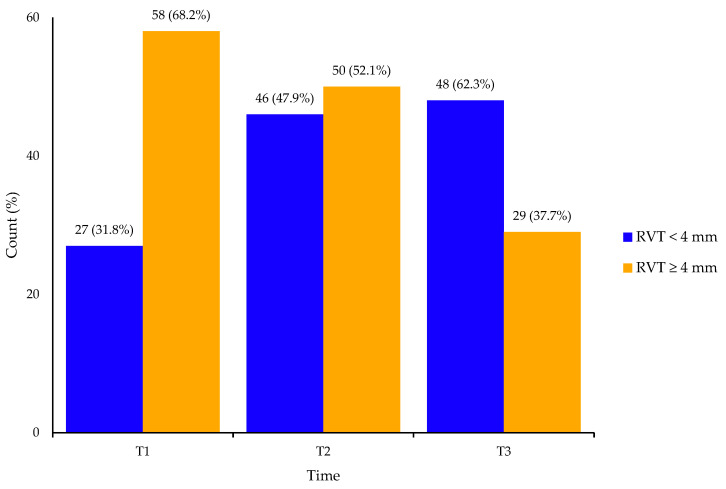
RVT rates at 6 weeks (T1), 3 months (T2) and 6 months (T3), showing progressive recanalization over time.

The prevalence of RVT ≥ 4 mm was 68.2% (58/85) at T1, 52.1% (50/96) at T2 and 37.7% (29/77) at T3 (Figure 1). Among the 50 patients with RVT at T2, 12 did not attend the T3 follow-up; thus, RVT persistence between T2 and T3 was 76.3% (29/38).

### 3.2. Therapeutic Decisions

At the end of the study, therapeutic strategies were individualized according to the balance between recurrence and bleeding risk: 62/96 patients (64.6%) continued full-dose anticoagulation, 20 (20.8%) continued at a reduced dose for secondary prevention, 11 (11.5%) discontinued at T3, 2 (2.1%) discontinued at T2 and 1 (1.0%) switched to another DOAC. Notably, the two patients who discontinued after 3 months had negative RVT at T2 and no additional recurrence risk factors. Among those completing the 6-month follow-up (*n* = 77), 50 (64.9%) continued full-dose therapy, 17 (22.1%) switched to a reduced dose and 10 (13.0%) discontinued treatment. Importantly, none of the patients with RVT ≥ 4 mm at T3 discontinued therapy: 23 (79.3%) continued at full dose, while 6 (20.7%) continued at a reduced dose. By contrast, among those with RVT < 4 mm, 10 (20.8%) discontinued therapy, 12 (25.0%) switched to a prophylactic dose and 26 (54.2%) remained on full-dose treatment, highlighting that RVT was only one of several factors influencing clinical decisions.

### 3.3. Safety Outcomes

No thrombotic recurrences occurred during the study. Regarding bleeding complications, there were eight cases of clinically relevant non-major bleeding (CRNMB), one case of major gastrointestinal bleeding requiring hospitalization and transfusion and one fatal intracranial hemorrhage. Table 3 summarizes the above-mentioned results.

### 3.4. Predictors of RVT

Table 4 shows the baseline characteristics of patients stratified by RVT status at T2. Male sex was significantly associated with RVT persistence: among patients with recanalization (RVT < 4 mm), 24/46 (52.2%) were women, whereas among those with RVT ≥ 4 mm, 41/50 (82.0%) were men (*p* = 0.001). No significant differences were observed between groups in terms of age (median 61.5 [47.0–73.8] vs. 64.0 [55.5–74.8] years, *p* = 0.346).

Smoking (21.7% vs. 20.0%, *p* = 1.000), diabetes (4.4% vs. 10.0%, *p* = 0.438), hypertension (52.2% vs. 46.0%, *p* = 0.689) and BMI (26.7 [23.1–31.1] vs. 26.1 [23.2–27.6], *p* = 0.215) were not significantly associated with RVT. Inherited/acquired thrombophilia also showed no significant association, although family history of thrombosis was strongly correlated: 28.0% of patients with RVT < 4 mm had a positive family history, compared with only 6.5% of those with RVT ≥ 4 mm (*p* = 0.013).

The extension of thrombosis correlated with RVT persistence. The involvement of the common femoral vein was more frequent in patients with RVT ≥ 4 mm (60.0% vs. 34.8%, *p* = 0.023), and combined femoropopliteal involvement was significantly associated with RVT (54.0% vs. 23.9%, *p* = 0.005). Iliac vein extension and bilateral thrombosis, however, were not statistically significant predictors (*p* = 0.266 and *p* = 1.000, respectively). DVT type (provoked vs. unprovoked vs. cancer-related) and concomitant PE were not significantly associated with RVT persistence.

### 3.5. Drugs Comparison

No significant differences were observed between Apixaban and Rivaroxaban in terms of recanalization (*p* = 0.635). The use of a loading dose did not influence RVT rates (50.0% vs. 43.5%, *p* = 0.922).

### 3.6. Multivariable Analysis

A logistic regression model (Table 5) was performed including variables significant in univariate analysis. To avoid multicollinearity, femoropopliteal involvement was included as a composite variable, excluding femoral vein involvement alone. Female sex was independently associated with a markedly reduced risk of RVT (OR = 0.20, 95% CI: 0.07–0.52), while femoropopliteal involvement (OR = 4.13, 95% CI: 1.58–11.7) and family history of VTE (OR = 4.58, 95% CI: 1.18–23.5) were independently associated with increased odds of RVT persistence at 3 months.

## 4. Discussion

Our study showed a progressively decreasing trend in RVT rates over time, from 68.2% at 6 weeks to 52.1% at 3 months and 37.7% at 6 months, in agreement with previously published studies [24,25,26,27]. For instance, Soares et al. prospectively investigated recanalization in 51 patients treated with Rivaroxaban, reporting partial recanalization rates of 71.7% at 3 months and 45% at 6 months. However, different methods of RVT assessment were used, which prevents a direct comparison with our findings [27]. In 2017, Prandoni et al. also evaluated residual thrombosis in a large Italian cohort treated with DOACs (mostly Rivaroxaban) and compared the results with historical data from patients on VKAs. That study showed a progressively higher rate of recanalization in patients receiving DOACs, with RVT rates slightly lower than in our study (where Rivaroxaban and Apixaban were almost equally represented), but reasonably comparable, despite the limitations arising from loss to follow-up in both cohorts [19].

Few data are available regarding the efficacy of Coumarins compared with DOACs. Recently, Erol et al. investigated factors influencing recanalization in a large cohort of patients treated with Warfarin or DOACs and found no significant differences between the two groups. However, that study was retrospective and included heterogeneous ultrasound time points; in addition, most patients in the DOAC group were treated with Rivaroxaban. Furthermore, the definition of RVT differed from ours [28].

In our cohort, the RVT persistence rate between T2 and T3 was 76.3%, confirming that most recanalization occurs within the first three months after the index event. Interestingly, we also observed that about 32% of patients achieved early recanalization within 45 days. Few studies have evaluated RVT at 1 month. Despite the lack of standardized criteria to define residual thrombosis, RVT was found in over 90% of unprovoked thromboses treated with VKAs and, more recently, also in patients treated with Rivaroxaban [11,29].

A systematic review and meta-analysis by Donadini et al., which included 2527 patients treated with VKAs, showed that RVT was only a weak predictor of recurrence when detected early (i.e., at 3 months) after unprovoked DVT, and that it lost prognostic significance when detected later [12]. Moreover, Prandoni et al. demonstrated that RVT at 3 months not only predicts recurrence but is also associated with long-term complications such as post-thrombotic syndrome [30]. Therefore, earlier recanalization, which appears to be favored by DOAC treatment, may contribute to the prevention of such outcomes.

When we analyzed the nature of DVT, we did not observe significant differences between groups: unprovoked and provoked DVT were not associated with different recanalization rates during DOAC treatment. This is consistent with Erol’s findings, where provoked DVT did not recanalize more efficiently, and with a previous study by Prandoni, which reported no significant difference in RVT rates between idiopathic and secondary thrombosis in patients on VKAs [10,28]. Our results reinforce the concept that, while RVT can reflect thrombotic burden, it cannot serve as the sole predictor of recurrence. Indeed, current clinical decisions on anticoagulation duration are made irrespective of whether DVT is provoked or unprovoked, as confirmed by the START VTE trial [31].

In the past, combining RVT with D-dimer has shown better predictive value for recurrence [10,16]. However, this was not one of our study endpoints, and the observational design did not allow for standardized laboratory data collection, particularly during the SARS-CoV-2 pandemic.

An encouraging finding is the high recanalization rate observed in cancer patients: despite the limited sample size, RVT was absent in two-thirds of cases. This rate was higher than that reported by Piovella and closer to that of Dentali in cancer-associated distal thrombosis treated with VKAs and heparins [11,29]. Although preliminary, these results suggest that the stable anticoagulation achievable with DOACs during chemotherapy—characterized by fewer drug–drug interactions, lower food interference, and limited hepatic metabolism—may represent an advantage. Moreover, in vitro studies have suggested that Apixaban and Rivaroxaban may enhance FX-dependent fibrinolysis and tissue plasminogen activator (tPA)-mediated plasmin generation [32]. Indeed, the role of endogenous tPA and plasminogen activator inhibitor (PAI) in thrombus resolution has been well documented [33,34].

We also explored whether DOAC treatment could influence recanalization in specific subgroups at risk of RVT persistence. Although the evidence remains debated, in our cohort male sex, femoral involvement, extensive thrombosis, and a family history of DVT were associated with reduced recanalization, consistent with findings in patients on VKAs. Conversely, obesity, personal history of thrombosis, diabetes, and inherited thrombophilia did not significantly affect RVT rates, suggesting a potentially protective effect of DOACs in these subgroups.

When comparing individual DOACs, our study did not identify any significant difference in efficacy: recanalization rates at 3 months were similar, and no effect was observed when the initial loading dose was considered as a variable.

Taken together, these considerations reinforce the need for further trials evaluating RVT during DOAC treatment, where early recanalization could represent a criterion for discontinuing anticoagulation. RVT could be incorporated into prediction models with standardized definitions and predefined assessment time points (e.g., at 1 and 3 months), to better identify patients with a favorable prognosis who may benefit from early anticoagulation discontinuation—a topic that remains highly relevant [35].

## 5. Conclusions

In the treatment of venous thromboembolism (VTE), it is widely recognized that a minimum of 3–6 months of anticoagulation is required to achieve adequate venous recanalization. By contrast, no universally adopted guidelines exist regarding secondary prevention, and the decision to extend anticoagulation must rely on careful individual risk assessment. Prolonging therapy effectively reduces recurrence risk, but the associated bleeding risk remains a major concern. The introduction of reduced-dose anticoagulant regimens has made long-term therapy safer, yet in certain high-risk patients, full-dose anticoagulation continues to represent the only viable option. Clinicians are therefore required to weigh multiple individual factors to establish personalized risk profiles. Within this framework, residual vein thrombosis (RVT) has remained an area of debate for more than three decades. Several studies have suggested an increased recurrence risk in patients with RVT meeting specific criteria, and RVT has consequently been incorporated into some management strategies [1,6,10,11,16,19,36]. Nevertheless, heterogeneity in assessment methods and the predominance of data from the Warfarin era limit its clinical applicability. Our study was designed in this context, during the DOAC era, to evaluate the effect of novel oral anticoagulants on RVT persistence—defined here as residual thrombus ≥ 4 mm—and to explore whether predictors of RVT are influenced by DOAC treatment. We observed that RVT rates progressively declined over time, from nearly two-thirds at six weeks, to one-half at three months and to one-third at six months. These results mirror recanalization patterns previously reported with VKAs, though methodological differences and distinct study populations preclude direct comparisons [10,11]. Notably, we also found evidence of an early effect of DOACs on recanalization, with significant improvements already evident at one month. Consistent with the prior literature, certain subgroups demonstrated poorer recanalization—specifically, male patients, those with proximal femoropopliteal thrombosis and those with a family history of VTE—while DOACs appeared to confer potential protective effects in others, such as patients with thrombophilia, obesity, prior DVT and particularly cancer.

Evidence comparing DOACs with Warfarin remains inconclusive. For instance, a retrospective study by Erol et al. evaluating 666 femoropopliteal thromboses reported no significant differences in recanalization between DOACs (mainly Rivaroxaban and Apixaban) and Warfarin [28]. 

In our study, no statistically significant differences emerged between Apixaban and Rivaroxaban, and efficacy appeared comparable regardless of whether a loading dose was used. Direct comparisons between these drugs are still very exiguous and with no univocal results [37,38,39]. To our knowledge, this represents the first prospective head-to-head comparison of Apixaban versus Rivaroxaban in patients with acute DVT. 

The main limitations of our study include the relatively small sample size—partially influenced by the SARS-CoV-2 pandemic—and its single-center design, which limit the generalizability of findings, particularly in subgroups such as patients with cancer. Further studies are needed to clarify the role of RVT in clinical practice and to determine whether DOACs can influence the natural history of venous recanalization. If the predictive value of RVT for recurrence is confirmed, extended anticoagulation may become the new standard of care, with the dual objective of enhancing recanalization and reducing recurrence rates.

## Figures and Tables

**Table 1:** The main demographic characteristics of the patients included in the statistical analysis at the time of diagnosis.

Total number of enrolled patients	113
Patients included in the statistical analysis	96
Gender, *n.* (%)	M 63 (65.6%); F 33 (34.4%)
Age, years old (mean ± SD)	61.9 ± 16.9
Age > 60 y.o., *n.* (%)	55 (57.3%)
Weight, Kg (mean ± SD)	78.6 ± 16.5
BMI, Kg/m^2^ (mean ± SD)	26.4 ± 5.1
Obese patients with BMI ≥ 30, *n.* (%)	19 (19.8%)
Weight in obese patients, Kg (mean ± SD)	95.6 ± 15.1
BMI in obese patients, Kg/m^2^ (mean ± SD)	33.8 ± 3.7
Personal history of VTE, *n.* (%)	10 (10.4%)
Family history of VTE, *n.* (%)	17 (17.7%)

SD: standard deviation; BMI: body mass index; VTE: venous thromboembolism.

**Table 2:** The main clinical characteristics of the patients included in the statistical analysis.

Site of DVT, *n.* (%)	- only PV, 50 (52.1%) - only CFV, 8 (8.3%) - PV and CFV, 38 (39.6%) - Bilateral, 7 (7.3%) - Iliac extension, 18 (18.8%)
Associated PE, *n.* (%)	36 (37.5%)
Nature of DVT	- unprovoked, 44 (45.8%) - provoked by weak risk factors, 31 (32.3%) - provoked by strong risk factors, 12 (12.5%) - cancer-associated, 9 (9.4%)
Time between symptoms onset and diagnosis, *n.* (%)	- <1 week, 43 (44.8%) - 1–4 weeks, 26 (27.1%) - >4 weeks, 15 (15.6%) - Unknown, 12 (12.5%)
Mean time to start DOACs	10 days
Full-dose DOACs, *n.* (%)	95 (99.0%)
Loading dose of DOACs, *n.* (%)	47 (49.0%)
Apixaban, *n.* (%)	50 (52.1%)
Rivaroxaban, *n.* (%)	42 (43.8%)
Edoxaban, *n.* (%)	3 (3.1%)
Dabigatran, *n.* (%)	1 (1.0%)
Inherited thrombophilia ^1^, *n.* (%)	22 (40.0%) ^2^
Smoke, *n.* (%)	20 (20.8%)
Alcohol abuse, *n.* (%)	20 (20.8%)
Active cancer, *n.* (%)	9 (9.4%)
Diabetes, *n.* (%)	7 (7.3%)
Recent surgery, *n.* (%)	4 (4.0%)
Bleeding history, *n.* (%)	7 (7.3%)
Chronic liver disease, *n.* (%)	3 (3.1%)
Renal failure, *n.* (%)	17 (17.7%)
Hypertension, *n.* (%)	47 (48.9%)
Previous stroke, *n.* (%)	5 (5.2%)
Frequent falls, *n.* (%)	0 (0%)
Chronic use of NSAIDs, *n.* (%)	8 (8.3%)

DVT: deep vein thrombosis; PV: popliteal vein; CFV: common femoral vein; PE: pulmonary embolism; DOACs: direct oral anticoagulants; NSAIDs: non-steroidal anti-inflammatory drugs. ^1^ Inherited thrombophilia was tested in 55 patients. ^2^ Low risk, 16 (29.1%); high risk, 4 (7.3%); antiphospholipid antibodies, 2 (3.6%).

**Table 3:** The main results and therapeutic strategies adopted.

Patients with T1, *n.* (%)	85 (88.5%)
Patients with T2, *n.* (%)	96 (100%)
Patients with T3, *n.* (%)	77 (80.2%)
RVT ≥ 4 mm, *n.* (%)	- T1, 58/85 (68.2%) - T2, 50/96 (52.1%) - T3, 29/77 (37.7%)
Overall therapeutic decision, *n.* (%)	- Prosecution at the same dose, 62/96 (64.6%) - Discontinuation, 13/96 (13.6%) ^1^ - Reduction to prophylactic dose, 20/96 (20.8%) - Switch to other DOAC, 1/96 (1.0%)
Therapeutic decision at T3, *n.* (%) ^2^	- Prosecution at the same dose, 50/77 (64.9%) - Discontinuation, 10/77 (13.0%) - Reduction to prophylactic dose, 17/77 (22.1%)
Thrombotic recurrence during the study, *n.* (%)	0 (0%)
Bleedings, *n.* (%)	- No, 86 (89.6%) - CRNMB, 8 (8.4%) - Major, 1 (1.0%) - Intracranial, 1 (1.0%)

RVT: residual vein thrombosis; DOAC: direct oral anticoagulant; CRNMB: clinically relevant non-major bleedings. ^1^ Two discontinued at T2 (2.1%); 11 discontinued at T3 (11.5%). ^2^ Including only patients who participated in the T3 control.

**Table 4:** Demographic, clinical and thrombotic characteristics of patients stratified by residual vein thrombosis (RVT < 4 mm vs. RVT ≥ 4 mm) at 3 months (T2). Data are reported as median [Q1; Q3] for continuous variables and as absolute frequency (percentage) for categorical variables. *p*-values refer to univariate comparisons (Pearson’s χ^2^ test or Mann–Whitney U test, as appropriate).

	RVT < 4 mm N = 46	RVT ≥ 4 mm N = 50	*p*-Value
Gender: F M	24 (52.2%) 22 (47.8%)	9 (18.0%) 41 (82.0%)	**0.001**
Age, years:	61.5 [47.0; 73.8]	64.0 [55.5; 74.8]	0.346
Smoking habit: No Yes	36 (78.3%) 10 (21.7%)	40 (80.0%) 10 (20.0%)	1.000
Diabetes: No Yes	44 (95.7%) 2 (4.3%)	45 (90.0%) 5 (10.0%)	0.438
Hypertension: No Yes	22 (47.8%) 24 (52.2%)	27 (54.0%) 23 (46.0%)	0.689
BMI, kg/m^2^	26.7 [23.1; 31.1]	26.1 [23.2; 27.6]	0.215
Thrombophilia: Absent Inherited, low risk ^1^ Inherited, high risk ^2^ APS Not tested	16 (34.8%) 8 (17.4%) 1 (2.2%) 1 (2.2%) 20 (43.4%)	17 (34.0%) 8 (16.0%) 3 (6.0%) 1 (2.0%) 21 (42.0%)	0.942
VTE familiar history: No Yes	43 (93.5%) 3 (6.5%)	36 (72.0%) 14 (28.0%)	**0.013**
VTE personal history: No Yes	41 (89.1%) 5 (10.9%)	45 (90.0%) 5 (10.0%)	1.000
Popliteal vein involvement: No Yes	5 (10.9%) 41 (89.1%)	3 (6.0%) 47 (94.0%)	0.474
Femoral vein involvement: No Yes	30 (65.2%) 16 (34.8%)	20 (40.0%) 30 (60.0%)	**0.023**
Femoropopliteal involvement: No Yes	35 (76.1%) 11 (23.9%)	23 (46.0%) 27 (54.0%)	**0.005**
Iliac vein involvement: No Yes	40 (87.0%) 6 (13.0%)	38 (76.0%) 12 (24.0%)	0.266
Bilateral involvement: No Yes	43 (93.5%) 3 (6.5%)	46 (92.0%) 4 (8.0%)	1.000
Nature of DVT: Idiopathic Associated with weak risk factors Associated with strong risk factors Cancer associated	17 (37.0%) 15 (32.6%) 8 (17.4%) 6 (13.0%)	27 (54.0%) 16 (32.0%) 4 (8.0%) 3 (6.0%)	0.217
Pulmonary embolism: No Yes	26 (56.5%) 20 (43.5%)	34 (68.0%) 16 (32.0%)	0.342
Drugs: Apixaban Rivaroxaban	25 (58.1%) 18 (41.9%)	25 (51.0%) 24 (49.0%)	0.635
Loading dose: No Yes	20 (43.6%) 23 (50.0%)	25 (50.0%) 24 (48.0%)	0.922

RVT: residual vein thrombosis; BMI: body mass index; APS: antiphospholipid syndrome; VTE: venous thromboembolism; DVT: deep vein thrombosis. ^1^ Including a single heterozygosity for prothrombin polymorphism or FV Leiden. ^2^ Including homozygosis or compound heterozygosis for prothrombin polymorphism or FV Leiden, protein S/C deficiency or antithrombin deficiency. Bold *p*-values are less than 0.05.

**Table 5:** Multivariable logistic regression analysis of the association between clinical characteristics and the presence of RVT at 3 months.

Characteristic	OR	95% CI	*p*-Value
Gender:			
M	—	—	
F	0.20	0.07, 0.52	0.002
Femoropopliteal involvement:			
No	—	—	
Yes	4.13	1.58, 11.7	0.005
VTE familiar history:			
No	—	—	
Yes	4.58	1.18, 23.5	0.040

OR: odds ratio; CI: confidence interval.

## Data Availability

Data are unavailable due to privacy reasons.

## Abbreviations

The following abbreviations are used in this manuscript:

READ	Residual vein thrombosis and DOACs
RVT	Residual vein thrombosis
RVO	Residual vein obstruction
DOACs	Direct oral anticoagulants
VKAs	Vitamin K antagonists
VTE	Venous thromboembolism
DVT	Deep vein thrombosis
PE	Pulmonary embolism
CUS	Compression ultrasonography
